# Comparing supervised and semi-supervised machine learning approaches in NTCP modeling to predict complications in head and neck cancer patients

**DOI:** 10.1016/j.ctro.2023.100677

**Published:** 2023-09-21

**Authors:** I. Spiero, E. Schuit, O.B. Wijers, F.J.P. Hoebers, J.A. Langendijk, A.M. Leeuwenberg

**Affiliations:** a Julius Center for Health Sciences and Primary Care, University Medical Center Utrecht, Utrecht University, Utrecht, the Netherlands; b Radiotherapeutic Institute Friesland, Leeuwarden, the Netherlands; c Department of Radiation Oncology (Maastro), GROW School for Oncology and Reproduction, Maastricht University Medical Centre+, Maastricht, the Netherlands; d Department of Radiation Oncology, University of Groningen, University Medical Center Groningen, Groningen, the Netherlands

**Keywords:** Head and neck cancer, Radiation-induced toxicity, NTCP modeling, Semi-supervised learning

## Abstract

**Background and purpose:**

Head and neck cancer (HNC) patients treated with radiotherapy often suffer from radiation-induced toxicities. Normal Tissue Complication Probability (NTCP) modeling can be used to determine the probability to develop these toxicities based on patient, tumor, treatment and dose characteristics. Since the currently used NTCP models are developed using supervised methods that discard unlabeled patient data, we assessed whether the addition of unlabeled patient data by using semi-supervised modeling would gain predictive performance.

**Materials and methods:**

The semi-supervised method of self-training was compared to supervised regression methods with and without prior multiple imputation by chained equation (MICE). The models were developed for the most common toxicity outcomes in HNC patients, xerostomia (dry mouth) and dysphagia (difficulty swallowing), measured at six months after treatment, in a development cohort of 750 HNC patients. The models were externally validated in a validation cohort of 395 HNC patients. Model performance was assessed by discrimination and calibration.

**Results:**

MICE and self-training did not improve performance in terms of discrimination or calibration at external validation compared to current regression models. In addition, the relative performance of the different models did not change upon a decrease in the amount of (labeled) data available for model development. Models using ridge regression outperformed the logistic models for the dysphagia outcome.

**Conclusion:**

Since there was no apparent gain in the addition of unlabeled patient data by using the semi-supervised method of self-training or MICE, the supervised regression models would still be preferred in current NTCP modeling for HNC patients.

## Highlights

• Supervised and semi-supervised machine learning approaches to predict toxicities in head and neck cancer patients were compared.
• Similar performance of the models was observed both in terms of discrimination and calibration.
• Varying the amount of data for development or the confidence threshold did not impact the similarity in performance.
• The (supervised and semi-supervised) models involving ridge regression outperformed the logistic regression models for the dysphagia outcomes.

## Introduction

Head and neck cancer (HNC) patients who undergo radiotherapy treatment often suffer from toxicities such as xerostomia (dry mouth) and dysphagia (difficulty swallowing) [1]. Normal Tissue Complication Probability (NTCP) modeling can inform clinical decision making to reduce these toxicities, by describing the relationship between dose and volume of irradiated normal tissues and the risk to develop toxicities [2]. Currently, such NTCP models are used to make predictions for HNC patients in the decision to assign them to a new radiotherapy using protons [3]. This new therapy can – for certain patients – reduce the risk of radiation-induced toxicities, but due to practical constraints such as higher costs and limited availability only a selective number of patients can be assigned to this therapy. Whether the expected reduction in complication risk is sufficiently lower for proton therapy is determined by using the modeled probabilities to compute the difference in the predicted risk between photon and proton therapy (see [3], [4] for further details).

Typically, NTCP models are regression models that only use patients from whom the toxicity outcomes have been recorded, the labeled data, to develop the model. However, as toxicities often arise long after the radiotherapy treatment has started [1], several unlabeled observations are also available, in which the patient’s background and treatment information has been documented, but not yet their toxicity outcomes. These unlabeled data are not used in regression models, but the information that is present in these observations may possibly be of use to improve NTCP model performance.

To include unlabeled data in NTCP modeling, semi-supervised learning techniques can be used, which can deal with both labeled and unlabeled data [5]. Depending on the specific dataset at hand, semi-supervised methods may improve or degrade model performance compared to their supervised equivalents [5], [6]. One widely known method within semi-supervised learning is self-training [7], [8]. Self-training is computationally efficient and easily interpretable, making the method attractive to apply in clinical prediction settings as opposed to the more advanced semi-supervised methods that are available. In self-training, the model iteratively trains itself on the labeled data using a supervised classifier to assign *predicted* outcome labels (called pseudolabels) to the unlabeled data. Each iteration, the model is re-trained using the labeled and pseudolabeled data [5].

Self-training has only once been evaluated previously within NTCP modeling for HNC patients, treated at the Portuguese Institute of Oncology of Coimbra [9]. The data contained 84 unlabeled observations of the 222 patients in total, and the use of self-training with a random forest classifier showed a gain in discrimination performance compared to supervised learning, though calibration performance was not reported. Here, it will be evaluated how the addition of unlabeled patient data by semi-supervised learning compares in performance to the current supervised regression methods in NTCP modeling for proton therapy selection, using a dataset of 750 HNC patients treated at the University Medical Center Groningen.

## Materials & methods

### Study design

The development cohort consisted of 750 HNC patients treated at the University Medical Center Groningen (between January 2007 and June 2016). The validation cohort consisted of 395 patients treated at the University Medical Center Groningen (between July 2016 and December 2017), Maastro Clinic (between May 2012 and June 2016), and Radiotherapeutic Institute Friesland (between May 2014 and December 2016). Both cohorts were treated with photon therapy and correspond to the development and validation cohorts of the NTCP models currently used in the Dutch National Indication Protocol Proton Therapy [4].

In both cohorts, the presence of toxicities in HNC patients was scored at different intervals during and after radiotherapy treatment. For each observation, the patient, tumor, and treatment characteristics and dose parameters of 28 organs were also included in the dataset. Further details on the content, collection, and inclusion requirements of the data have been previously described [10].

### Missing data

Missing data were present in baseline toxicity scores and toxicity outcomes as described in Table 1. Multiple imputation by chained equations (MICE) was used to impute the missing data, assuming that the missing data were missing at random. The data were imputed separately in the development (once) and in the validation cohort (ten times, also separately per center) following [10]. The multiple imputed datasets were used in further analyses and the results were pooled according to Rubin’s rules [11].

**Table 1:** Description of the development and validation cohorts.

	**Development cohort (n = 750)**		**Validation cohort (n = 395)**	
		Missing (%):		Missing (%):
**Patient** Mean age (sd) Sex (%) Male Female Tumor stage (%) Tis-T2 T3-T4	63.1 (10.2) 560 (75%) 190 (25%) 363 (48%) 387 (52%)	- - - - -	64 (9.4) 290 (73%) 105 (27%) 194 (49%) 201 (51%)	- - - - -
**Mean dose (Gy) to the (sd)** Submandibular glands Parotid glands Pharyngeal constrictor muscle (PCM) superior Pharyngeal constrictor muscle (PCM) medius Pharyngeal constrictor muscle (PCM) inferior	48.5 (22.9) 51.7 (32.0) 42.9 (24.1) 48.7 (20.3) 54.7 (13.0)	2 (0.3%) - - - -	44.7 (20.7) 51.7 (32.0) 38.7 (23.) 49.4 (20.1) 53.0 (14.1)	- - - - -
**Toxicity at baseline (%)** Xerostomia grade ≥ 2 Xerostomia grade ≥ 3 Dysphagia grade ≥ 2 Dysphagia grade ≥ 3	75 (11%) 16 (2%) 178 (24%) 62 (8%)	85 (11%) 85 (11%) 14 (2%) 14 (2%)	52 (18%) 20 (7%) 85 (22%) 21 (5%)	99 (25%) 99 (25%) - -
**Toxicity at 6 months (%)** Xerostomia grade ≥ 2 Xerostomia grade ≥ 3 Dysphagia grade ≥ 2 Dysphagia grade ≥ 3	260 (44%) 78 (13%) 183 (29%) 94 (15%)	160 (21%) 160 (21%) 118 (16%) 118 (16%)	93 (48%) 30 (15%) 61 (19%) 21 (6%)	201 (51%) 201 (51%) 71 (18%) 71 (18%)
**Primary tumor location** Pharynx Larynx	372 (50%) 334 (45%)	– -	205 (52%) 168 (43%)	– -

### Outcomes

We focused on the most common toxicities present at six months after the end of treatment, which are xerostomia (dry mouth) and dysphagia (difficulty swallowing). Xerostomia is a patient-reported item ranging from grade 1 to 4 (1 = “not at all”, 2 = “a little”, 3 = “quite a bit”, 4 = “very much”) based on EORTC QLQ-H&N35 (question 41). Dysphagia is a physician-rated item ranging from grade 1 to 5 (1 = “symptomatic but normal diet”, 2 = “only soft food”, 3–5 = “liquid food or tube feeding”) based on CTCAEv4.0 [4], [10]. A grade larger than 2 is considered as clinically relevant based on the severity of impact on a patient’s quality of life. The two toxicity outcomes were both dichotomized in two ways (grade ≥ 2 and grade ≥ 3), leading to a total of four outcomes that were modeled separately:

(a) xerostomia grade ≥ 2 (quite a bit or very much),
(b) xerostomia grade ≥ 3 (very much),
(c) dysphagia grade ≥ 2,
(d) dysphagia grade ≥ 3.

Patients who started their radiotherapy treatment shorter than six months ago are in practice unlabeled in the dataset, equal to approximately 40 patients. Therefore, to test the effect of unlabeled data in the practical setting of NTCP modeling, 40 random patient outcomes were made unlabeled.

### Predictors

In Table 2 the predictors are listed for the xerostomia (grade ≥ 2 and grade ≥ 3) and dysphagia (grade ≥ 2 and grade ≥ 3) models respectively. These predictors, and their transformations, correspond to the predictors included in the models described in the Dutch National Indication Protocol Proton Therapy [4], and had originally been selected from a set of candidate predictors based on prior knowledge and clinical expertise that was further examined for association with the toxicities by [10].

**Table 2:** The preselected set of predictors from the dataset that were used in the models to predict the presence of grade ≥ 2 and grade ≥ 3 xerostomia and dysphagia at 6 months after the end of radiotherapy in HNC patients.

**Predictors for xerostomia (grade ≥ 2 or 3)**	**Predictors for dysphagia (grade ≥ 2 or 3)**
Mean dose (Gy) to the (continuous) Submandibular glands Ipsilateral parotid gland (sqrt) + contralateral parotid gland (sqrt)	Mean dose (Gy) to the (continuous) Oral cavity Pharyngeal constrictor muscle (PCM) superior Pharyngeal constrictor muscle (PCM) medius Pharyngeal constrictor muscle (PCM) inferior
Xerostomia at baseline (binary) Grade ≥ 2 Grade ≥ 3	Dysphagia at baseline (binary) Grade ≥ 2 Grade ≥ 3
	Primary tumor location (binary) Pharynx Larynx

### Models

Six different models were created to compare the performance of supervised and semi-supervised methods and the inclusion of unlabeled data: (1) Logistic regression and (2) ridge regression were used as supervised baseline models. These two models could only use the part of the development cohort that is labeled (i.e., the development cohort minus the observations of six months that were made unlabeled). (3) Logistic regression and (4) ridge regression after multiple imputation of the 40 unlabeled outcomes with MICE were also developed. With these methods, a fully labeled dataset was created by MICE and used in the regression models. In this way, the methods are still being regarded as supervised methods. (5) Self-training with logistic regression as classifier and (6) self-training with ridge regression as classifier were used to examine whether the inclusion of unlabeled data by semi-supervised learning would improve NTCP modeling. The confidence threshold of the self-training was set at an intermediate value of 0.8, following Soares et al. [9]. The iterative process of self-training for model development and using the predictions to add pseudolabels was iterated until it reached a stopping criterion, which was determined to be when no unlabeled data were left or after a maximum of 50 iterations. Further details on the implementation of self-training can be found in Appendix B. These six models were developed separately for all four toxicity outcomes, leading to a total of 24 models.

### Model performance

To assess how well the models performed in patients not used for model development we externally validated the models using the validation cohort of 395 labeled observations. Both the discrimination (the ability to differentiate between patients with and without the outcomes) and the calibration (the consistency between predicted and actual probability of the outcome) of the models was evaluated, following [12] and [13] for the evaluation of prediction models. Calibration is important for ensuring that the predicted risks are reliable, and at external validation, this can be assessed by the calibration curve, intercept, and slope [14]. For the current setting of NTCP models for HNC patients, these measures are also known to have an impact on the difference in predicted risk of toxicity between photon and proton therapy, and therefore also on patient therapy selection [15].

For the discrimination, the area under the ROC curve (AUC) was used. The AUC lies between 0 and 1 with a higher AUC value indicating better discrimination of the model, and an AUC of 0.5 indicating no discriminative performance. For the calibration, the following measures were determined:

(1) ‘mean calibration’ (or ‘calibration-in-the-large’) which is the average predicted probability minus the overall outcome rate,
(2) ‘weak calibration’ by calculating the calibration slope, and
(3) ‘moderate calibration’ which visually shows how the estimated probabilities correspond to observed proportions with a calibration curve (closeness to the diagonal).

At external validation, these measures indicate whether the model has good generalizability (i.e. the ability to accurately predict outcomes for HNC patients from different but related populations).

### Ratio labeled vs. unlabeled data

In addition, it was evaluated whether differences in performance within or between the different models are dependent on the number of labeled observations in the dataset, since the performance of a model that is based on a smaller labeled training set is potentially more affected by the addition of pseudolabels. Therefore, in a separate analysis the number of observations was decreased stepwise. It was determined to keep the introduction of unlabeled data fixed at the average number of new patients each six months, which equals approximately 40 patients in this dataset. The amount of labeled data was then decreased stepwise by this number, by each time additionally removing 40 random observations from the dataset (Table A1).

After each decrease in number of observations in the dataset, all six models were again independently developed and externally validated for the four outcomes. For the regular logistic and ridge regression models (model 1 and 2), this meant that only the labeled observations that were left, were included in the development of the model. The other models did include the 40 unlabeled observations, either by imputation after MICE (model 3 and 4) or by pseudolabelling with self-training (model 5 and 6).

### Implementation details

Analyses were conducted in R (version 4.3.1). MICE imputation was performed using the 'mice' package (version 3.16.0) [16]. The val.prob.ci.2() function [17] was used to derive the evaluation measures.

## Results

The six models were externally validated in the validation cohort (n = 395). The calibration curves and the regression coefficients of all models for the four outcomes are presented in Appendix Figs. A3-A6 and Tables A2-A5, respectively. The number of iterations and pseudolabels added by the two self-training models are presented in Appendix Tables A6-A9.

### Model performances

The discrimination and calibration at external validation of the different models with 710 labeled and 40 unlabeled observations are presented in Table 3. Per toxicity outcome, the different models appear to show similar discriminative performance. For all but the xerostomia *≥ 3 outcome*, the three ridge regression models show values for calibration-in-the-large that are slightly closer to the ideal value of zero. Additionally, for all outcomes, the ridge regression models show values for the calibration slope that are closer to the ideal value of 1, as opposed to the three logistic regression models. The similarity of the six models is also apparent in the calibration curves (Appendix Figs. A3-A6), in which the shape and closeness to the diagonal of the curves are similar across the logistic regression models, and across the ridge regression models.

**Table 3:** External validation of the models using 710 labeled and 40 unlabeled observations for each of the four toxicity outcomes.

	**AUC (standard error)**	**Calibration-in-the-large (standard error)**	**Slope (standard error)**
**Xerostomia grade ≥ 2**			
Logistic regression	0.68 (0.027)	0.20 (0.109)	0.81 (0.134)
Ridge regression	0.68 (0.027)	0.18 (0.108)	0.89 (0.146)
MICE (logistic regression)	0.68 (0.027)	0.19 (0.110)	0.78 (0.131)
MICE (ridge regression)	0.68 (0.027)	0.17 (0.108)	0.87 (0.143)
Self-training (logistic regression)	0.68 (0.027)	0.20 (0.109)	0.80 (0.132)
Self-training (ridge regression)	0.68 (0.027)	0.18 (0.108)	0.89 (0.145)
**Xerostomia grade ≥ 3**			
Logistic regression	0.69 (0.035)	0.26 (0.141)	0.83 (0.184)
Ridge regression	0.70 (0.035)	0.29 (0.140)	0.93 (0.202)
MICE (logistic regression)	0.69 (0.035)	0.27 (0.141)	0.83 (0.184)
MICE (ridge regression)	0.70 (0.034)	0.30 (0.140)	0.93 (0.202)
Self-training (logistic regression)	0.69 (0.035)	0.30 (0.141)	0.83 (0.181)
Self-training (ridge regression)	0.70 (0.034)	0.33 (0.139)	0.99 (0.213)
**Dysphagia grade ≥ 2**			
Logistic regression	0.74 (0.026)	0.60 (0.134)	0.60 (0.086)
Ridge regression	0.74 (0.026)	0.23 (0.128)	0.75 (0.108)
MICE (logistic regression)	0.74 (0.026)	0.50 (0.134)	0.60 (0.087)
MICE (ridge regression)	0.74 (0.026)	0.20 (0.128)	0.74 (0.108)
Self-training (logistic regression)	0.74 (0.026)	0.63 (0.135)	0.59 (0.084)
Self-training (ridge regression)	0.74 (0.026)	0.25 (0.128)	0.74 (0.107)
**Dysphagia grade ≥ 3**			
Logistic regression	0.74 (0.038)	0.50 (0.181)	0.46 (0.097)
Ridge regression	0.72 (0.040)	0.08 (0.173)	0.55 (0.119)
MICE (logistic regression)	0.73 (0.037)	0.56 (0.180)	0.45 (0.098)
MICE (ridge regression)	0.72 (0.039)	0.14 (0.172)	0.56 (0.123)
Self-training (logistic regression)	0.74 (0.038)	0.58 (0.182)	0.45 (0.095)
Self-training (ridge regression)	0.72 (0.040)	0.11 (0.174)	0.54 (0.118)

### Model performances with decreasing data

**Fig. 1:**
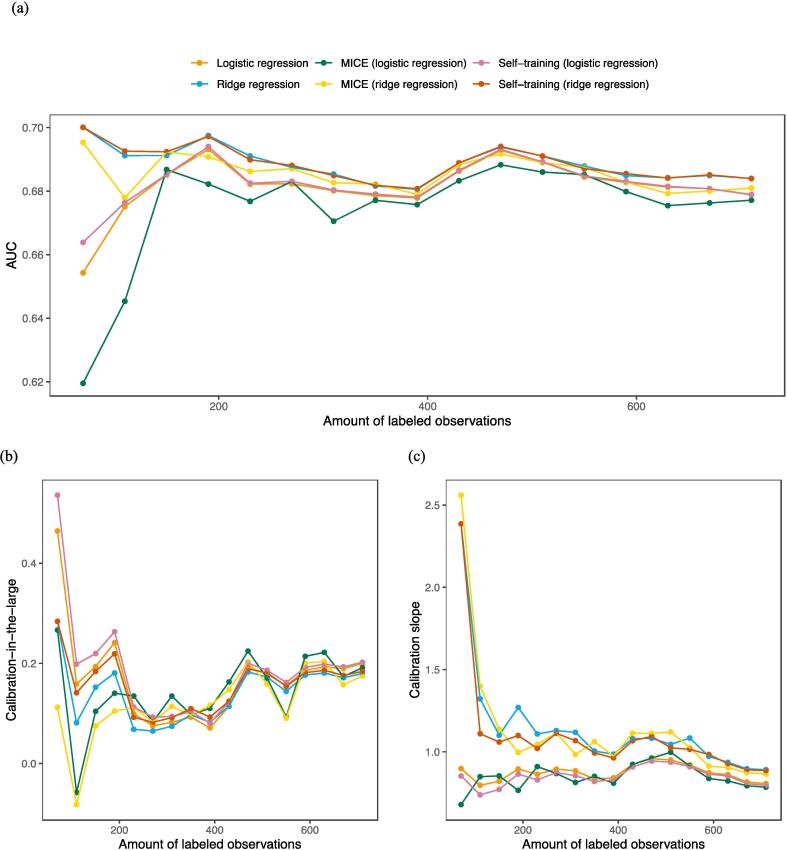
External validation of the models for xerostomia grade ≥ 2 for different amounts of labeled data. The amount of unlabeled data is fixed at 40 observations. (a) The AUCs, (b) the calibration intercepts, and (c) the calibration slopes.

**Fig. 2:**
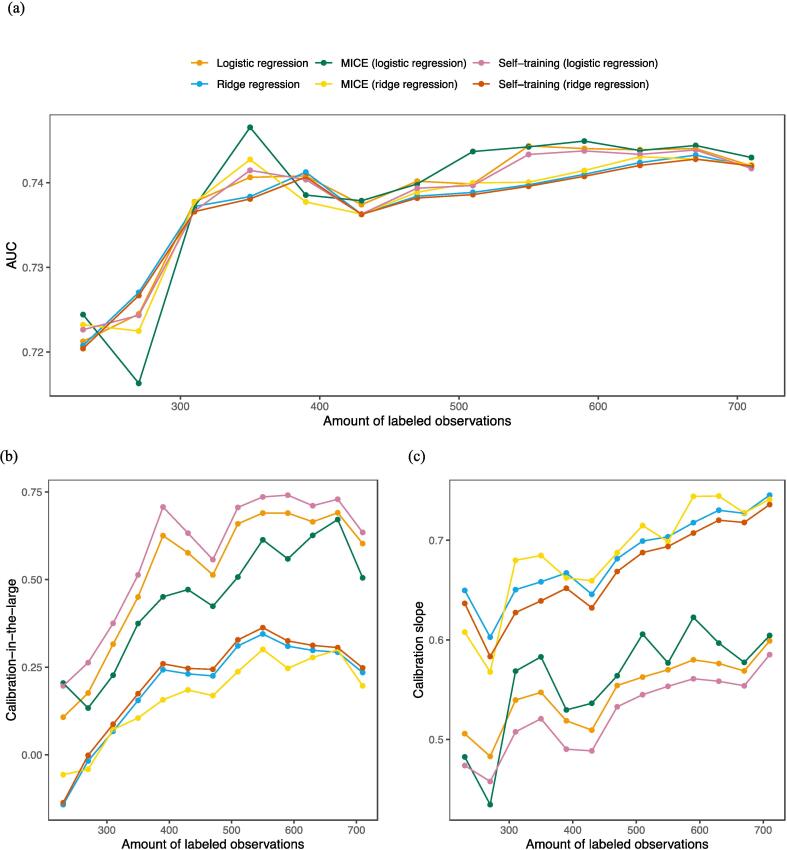
External validation of the models for dysphagia grade ≥ 2 for different amounts of labeled data. The amount of unlabeled data is fixed at 40 observations. (a) The AUCs, (b) the calibration intercepts, and (c) the calibration slopes.

To test whether the performance of the six models is dependent on the amount of data, the number of labeled observations in the dataset was decreased by steps of 40 observations. In Fig. 1, Fig. 2, the performance of the grade ≥ 2 models for the different amounts of labeled data are presented (the grade ≥ 3 models are presented in Appendix Figs. A1, A2). It is shown that the discriminative performance indicated by the AUC decreases when less labeled observations are used, except for the xerostomia ≥ 2 models where the AUC remains relatively stable. Furthermore, there are no clear differences in AUC between the six models for each of the outcomes. At very low amounts of labeled data for both xerostomia outcomes, the three ridge models maintain performance while the logistic models fail, and for both dysphagia outcomes the three ridge models have slightly lower AUC values, though these patterns are minimal. With regard to the calibration of the models, the models for xerostomia grade ≥ 2 and ≥ 3 do not clearly differ in calibration-in-the-large and calibration slope. However, for the dysphagia grade ≥ 2 and ≥ 3 models, there is a clear distinction in calibration between the logistic and the ridge regression models. Overall, the ridge models have calibration-in-the-large values closer to 0, and calibration slopes closer to 1. This pattern is most clear for the dysphagia ≥ 2, while for the dysphagia ≥ 3 outcome this also depends on the amount of labeled data.

### Self-training: Confidence threshold

As no substantial differences were found between the models with or without self-training, we decided to test the effect of the confidence threshold on the performance of the self-training method in this dataset. Therefore, self-training with logistic regression was repeated in an additional analysis for the xerostomia grade ≥ 2 outcome using confidence thresholds of 0.5, 0.6, 0.7, 0.8, 0.9, and 0.95, and then compared to regular logistic regression and logistic regression after MICE. It was found that with lower confidence thresholds, more pseudolabels were added during the self-training process, and for confidence thresholds above 0.8 only few of the 40 unlabeled data were pseudolabeled to almost none when the confidence threshold was set at 0.9 or larger (Appendix Table A10). However, with regard to the performance of the self-training at external validation, none of the tested thresholds used in self-training was related to a better performance compared to the regular logistic regression method (Appendix Fig. A7).

### Self-training: Ratio of labeled/unlabeled data

Since the number of 40 unlabeled observations that were introduced may be relatively low, we decided to examine the effect of larger proportions of unlabeled observations on the performance of the six different methods. In another additional analysis, the number of labeled observations was decreased by steps of 40, while the total number of observations was kept at 750. It was found that when the proportion of unlabeled data in the dataset was larger, the performance of the two self-training methods was slightly better in terms of discrimination (AUC), but worse in terms of calibration (calibration-in-the-large and calibration slope) compared to the other four methods when externally validated (Appendix Fig. A8). The calibration performance of the two self-training methods started to decrease below 600 observations, the point at which the methods started to add incorrectly classified pseudolabels (Appendix Table A11).

## Discussion

The aim of this study was to compare the currently used regression methods in NTCP modeling to the semi-supervised method of self-training and to regression after MICE, in order to examine the possible gain in performance when additionally using unlabeled data in model development. The results show that none of the six tested models had the best discrimination (AUC) and calibration (calibration-in-the-large and calibration slope) across all separately modeled outcomes (xerostomia ≥ grade 2, xerostomia ≥ grade 3, dysphagia grade ≥ 2, dysphagia grade ≥ 3) when externally validated. Across different amounts of labeled data, self-training with logistic regression or ridge regression tended to perform similarly or even slightly worse than the regular regression models. However, overall the three models based on ridge regression showed slightly better performance for the dysphagia outcomes.

Whether self-training would gain in model performance compared to supervised learning, depends on the dataset and specific research question at hand [5]. In current NTCP modeling, relatively few of the toxicity outcomes are unlabeled as only for most recent patients the toxicity outcomes have not yet been documented, which in this case is equal to about 40 patients within a dataset that has already over 700 labeled observations. This relatively small amount of unlabeled data may be the reason that self-training did not show better calibration or discrimination in this dataset. In the study by Soares et al. [9], self-training did show a gain in discriminative performance when the dataset had a larger proportion of unlabeled data (87 out of 222 observations), and also Chi et al. [18] found better discrimination and calibration performance when the amount of unlabeled data increased in a dataset of over 100.000 observations of the survival of colorectal cancer patients. The additional analysis in the current study also showed that when the proportion of unlabeled data was larger, the self-training methods performed slightly better in terms of discrimination, but not calibration. This may be due to the addition of incorrect pseudolabels that does not affect the model’s ability to discriminate between having the event or not, but may affect the exact predicted probabilities. The practical application of self-training may therefore be dependent on the proportion of labeled and unlabeled data at hand. Nevertheless, larger proportions of unlabeled data are not representative for the current practical setting of NTCP modeling for HNC patient selection.

More important than the number of unlabeled data included in the dataset, may be the information that is conveyed by the unlabeled observations [5], and the distribution of the examples in the classification problem [6]. When more overlap between the two classes of the outcome is present, the self-training may have more difficulty providing the correct pseudolabels and may therefore be impaired in its performance. This may have caused the similarities and degradations in performance of the self-training models compared to the logistic regression models in the current dataset.

An increase in the confidence threshold could prevent the addition of wrong pseudolabels, as the choice of the confidence threshold can significantly influence the performance of self-training [5]. For the xerostomia grade ≥ 2 outcome in the current dataset, the confidence threshold determined how many (incorrect) pseudolabels were added, but not how well the model performed. It could be further examined whether changes in the confidence threshold of the self-training method would improve the performance of the self-training for different outcomes, and additionally, if and how the optimal confidence threshold varies for different (number of events in the) outcomes.

Even though self-training showed no gain in performance for this dataset, the three models that used ridge regression did sometimes show a better performance compared to the three logistic models. Especially for the dysphagia models, for which the number of predictors was larger (eight for dysphagia compared to four for xerostomia), this difference was more apparent. Ridge regression aims to prevent overfitting by correcting for optimism with larger number of predictors and deals with multicollinearity [19], which is likely more applicable for the dysphagia predictors, but this was not further examined.

## Conclusion

In conclusion, the addition of unlabeled data in NTCP modeling by semi-supervised self-training did not lead to a better performance in terms of discrimination and calibration. Furthermore, it was shown that for the xerostomia grade ≥ 2 outcome the confidence threshold of the self-training was not related to the performance of the self-training, but an increase in the ratio of unlabeled data did lead to a slightly better discriminative performance. Because the regression methods are most easily interpretable and applicable, and since self-training did not show a clear gain in performance, regression may be favored in practice.

## Patient consent statement

As the Dutch Medical Research Involving Human Subjects Act is not applicable to data collection as part of routine clinical practice, the requirement of informed consent was waived by the ethics committee.

## Author contributions

IS, ES, and AL contributed to the study design, the methodology and the conduct of the simulation studies. JL, OW, and FH provided the study data. IS drafted the first version of the manuscript, and all authors contributed to the writing and approval of the final version.

## Declaration of Competing Interest

The authors declare the following financial interests/personal relationships which may be considered as potential competing interests: J.A. Langendijk reports a relationship with Dutch Cancer Society: funding grants. J.A. Langendijk reports his department has research contracts with IBA, RaySearch, Siemens, Elekta, Leoni, and Mirada. J.A. Langendijk reports a relationship with Global Scientific Advisory Board of IBA, RayCare International Advisory Board of RaySearch that includes: board membership, consulting or advisory, and speaking and lecture fees. J.A. Langendijk reports a relationship with Netherlands Society for Radiation Oncology that includes: board membership.

*Funding*

AL is funded by the European Union’s Horizon 2020 research and innovation programme under grant agreement N° 825162. This dissemination reflects only the author's view and the Commission is not responsible for any use that may be made of the information it contains.
