# Supplementary material for: Comparing supervised and semi-supervised machine learning approaches in NTCP modeling to predict complications in head and neck cancer patients
Source: Clin Transl Radiat Oncol. 2023 Sep 21;43:100677. doi: 10.1016/j.ctro.2023.100677 (PMC10562149; doi:10.1016/j.ctro.2023.100677)
Supplement: Supplementary data 1 [file mmc1.pdf]

Supplementary material

Comparing supervised and semi-supervised machine learning approaches in NTCP modeling to predict complications in head and neck cancer patients

I. Spiero[a]*, E. Schuit[a], O.B. Wijers[b], F.J.P. Hoebers[c], J.A. Langendijk[d], A.M. Leeuwenberg[a]

[a] Julius Center for Health Sciences and Primary Care, University Medical Center Utrecht, Utrecht University, Utrecht, The Netherlands
[b] Radiotherapeutic Institute Friesland, Leeuwarden, The Netherlands
[c] Department of Radiation Oncology (Maastro), GROW School for Oncology and Reproduction, Maastricht University Medical Centre+, Maastricht, The Netherlands
[d] Department of Radiation Oncology, University of Groningen, University Medical Center Groningen, Groningen, The Netherlands

Appendix A

Table A1 | Amounts of labeled and unlabeled observations to compare the performance of the methods at different amounts of labeled data, including the corresponding absolute number of events and events per variable (EPV) for each of the four modeled outcomes respectively.

	**Xerostomia grade ≥ 2**		**Xerostomia grade ≥ 3**		**Dysphagia grade ≥ 2**		**Dysphagia grade ≥ 3**	
	Events	EPV	Events	EPV	Events	EPV	Events	EPV
710 labeled + 40 unlabeled	311	78	91	23	226	28	121	15
670 labeled + 40 unlabeled	297	74	87	22	213	27	115	14
630 labeled + 40 unlabeled	276	69	81	20	198	25	104	13
590 labeled + 40 unlabeled	257	64	75	19	187	23	100	13
550 labeled + 40 unlabeled	244	61	72	18	173	22	95	12
510 labeled + 40 unlabeled	226	57	69	17	163	20	88	11
470 labeled + 40 unlabeled	208	52	63	16	156	20	85	11
430 labeled + 40 unlabeled	193	48	54	14	145	18	78	10
390 labeled + 40 unlabeled	176	44	53	13	134	17	71	9
350 labeled + 40 unlabeled	159	40	49	12	123	15	65	8
310 labeled + 40 unlabeled	143	36	45	11	113	14	59	7
270 labeled + 40 unlabeled	126	32	39	10	101	13	53	7
230 labeled + 40 unlabeled	108	27	31	8	88	11	45	6
190 labeled + 40 unlabeled	85	21	-	-	-	-	-	-
150 labeled + 40 unlabeled	68	17	-	-	-	-	-	-
110 labeled + 40 unlabeled	52	13	-	-	-	-	-	-
70 labeled + 40 unlabeled	29	7	-	-	-	-	-	-

Table A2 | Regression coefficients of the models for xerostomia grade ≥ 2.

	(Intercept)	Mean dose to ipsilateral parotid (sqrt) + mean dose to contralateral parotid (sqrt)	Mean dose to submandibulars	Xerostomia at baseline grade ≥ 2	Xerostomia at baseline grade ≥ 3
Logistic regression	-2.605	0.157	0.011	0.502	1.078
Ridge regression	-2.425	0.121	0.015	0.44	0.965
MICE (logistic regression)	-2.612	0.149	0.012	0.54	1.177
MICE (ridge regression)	-2.435	0.118	0.015	0.474	1.058
Self-training (logistic regression)	-2.635	0.159	0.011	0.507	1.093
Self-training (ridge regression)	-2.446	0.121	0.015	0.444	0.968

Table A3 | Regression coefficients of the models for xerostomia grade ≥ 3.

	(Intercept)	Mean dose to ipsilateral parotid (sqrt) + mean dose to contralateral parotid (sqrt)	Mean dose to submandibulars	Xerostomia at baseline grade ≥ 2	Xerostomia at baseline grade ≥ 3
Logistic regression	-3.826	-0.059	0.042	0.001	1.128
Ridge regression	-3.639	0.008	0.027	-0.008	1.094
MICE (logistic regression)	-3.82	-0.058	0.042	-0.055	1.11
MICE (ridge regression)	-3.632	0.007	0.027	-0.059	1.072
Self-training (logistic regression)	-3.871	-0.044	0.039	0.029	1.206
Self-training (ridge regression)	-3.475	0.038	0.017	-0.007	1.081

Table A4 | Regression coefficients of the models for dysphagia grade ≥ 2.

	**(Intercept)**	**Mean dose to oral cavity**	**Mean dose to PCM superior**	**Mean dose to PCM medius**	**Mean dose to PCM inferior**	**Dysphagia at baseline grade ≥ 2**	**Dysphagia at baseline grade ≥ 3**	**Primary tumor location pharynx**	**Primary tumor location larynx**
Logistic regression	-5.439	0.085	-0.005	0.003	0.019	0.914	1.028	-0.358	-0.279
Ridge regression	-3.906	0.036	0.015	0.011	0.005	0.957	1.146	-0.289	-0.532
MICE (logistic regression)	-5.119	0.079	-0.007	0.013	0.014	0.957	1.038	-0.561	-0.553
MICE (ridge regression)	-3.823	0.035	0.014	0.013	0.004	0.989	1.146	-0.349	-0.597
Self-training (logistic regression)	-5.588	0.088	-0.006	0.002	0.019	0.927	1.078	-0.318	-0.214
Self-training (ridge regression)	-3.946	0.036	0.016	0.011	0.005	0.965	1.166	-0.281	-0.529

Table A5 | Regression coefficients of the models for dysphagia grade ≥ 3.

	**(Intercept)**	**Mean dose to oral cavity**	**Mean dose to PCM superior**	**Mean dose to PCM medius**	**Mean dose to PCM inferior**	**Dysphagia at baseline grade ≥ 2**	**Dysphagia at baseline grade ≥ 3**	**Primary tumor location pharynx**	**Primary tumor location larynx**
Logistic regression	-10.61	0.094	-0.014	0.032	0.05	0.357	1.367	0.52	-0.216
Ridge regression	-7.684	0.037	0.016	0.021	0.036	0.506	1.532	0.25	-0.631
MICE (logistic regression)	-10.266	0.099	-0.014	0.03	0.046	0.264	1.127	0.253	-0.369
MICE (ridge regression)	-7.395	0.04	0.017	0.02	0.031	0.447	1.352	0.081	-0.681
Self-training (logistic regression)	-10.885	0.099	-0.015	0.028	0.053	0.354	1.409	0.616	-0.122
Self-training (ridge regression)	-7.8	0.038	0.016	0.021	0.036	0.525	1.561	0.28	-0.611

Table A6 | The total number of pseudolabeled observations (and the number of which are incorrect) and number of iterations in which new labels were added of the self-training method with logistic regression and ridge regression respectively, for each dataset with decreasing numbers of labeled observations of xerostomia grade ≥ 2.

	**Self-training with logistic regression**		**Self-training with ridge regression**	
	Pseudolabels (incorrect)	Iterations	Pseudolabels (incorrect)	Iterations
710 labeled / 40 unlabeled	5 (0)	1	4 (0)	1
670 labeled / 40 unlabeled	5 (0)	1	4 (0)	1
630 labeled / 40 unlabeled	4 (0)	1	4 (0)	1
590 labeled / 40 unlabeled	4 (0)	1	4 (0)	1
550 labeled / 40 unlabeled	4 (0)	1	4 (0)	1
510 labeled / 40 unlabeled	4 (0)	1	4 (0)	1
470 labeled / 40 unlabeled	4 (0)	1	4 (0)	1
430 labeled / 40 unlabeled	4 (0)	1	4 (0)	1
390 labeled / 40 unlabeled	4 (0)	1	4 (0)	1
350 labeled / 40 unlabeled	4 (0)	1	4 (0)	1
310 labeled / 40 unlabeled	5 (0)	2	4 (0)	1
270 labeled / 40 unlabeled	4 (0)	1	4 (0)	1
230 labeled / 40 unlabeled	5 (0)	1	4 (0)	1
190 labeled / 40 unlabeled	4 (0)	1	4 (0)	1
150 labeled / 40 unlabeled	5 (0)	1	4 (0)	1
110 labeled / 40 unlabeled	5 (0)	1	4 (0)	3
70 labeled / 40 unlabeled	4 (0)	1	0 (0)	0

Table A7 | The total number of pseudolabeled observations (and the number of which are incorrect) and number of iterations in which new labels were added of the self-training method with logistic regression and ridge regression respectively, for each dataset with decreasing numbers of labeled observations of xerostomia grade ≥ 3.

	**Self-training with logistic regression**		**Self-training with ridge regression**	
	Pseudolabels (incorrect)	Iterations	Pseudolabels (incorrect)	Iterations
710 labeled / 40 unlabeled	32 (2)	1	32 (2)	1
670 labeled / 40 unlabeled	33 (3)	2	32 (2)	1
630 labeled / 40 unlabeled	32 (3)	2	32 (2)	1
590 labeled / 40 unlabeled	33 (3)	2	32 (2)	1
550 labeled / 40 unlabeled	31 (3)	2	32 (2)	1
510 labeled / 40 unlabeled	29 (3)	2	33 (3)	1
470 labeled / 40 unlabeled	30 (4)	2	34 (3)	2
430 labeled / 40 unlabeled	32 (5)	2	34 (3)	1
390 labeled / 40 unlabeled	31 (4)	3	33 (3)	2
350 labeled / 40 unlabeled	31 (4)	3	34 (3)	2
310 labeled / 40 unlabeled	29 (3)	2	33 (3)	2
270 labeled / 40 unlabeled	32 (4)	3	34 (3)	2
230 labeled / 40 unlabeled	30 (3)	1	32 (2)	1

Table A8 | The total number of pseudolabeled observations (and the number of which are incorrect) and number of iterations in which new labels were added of the self-training method with logistic regression and ridge regression respectively, for each dataset with decreasing numbers of labeled observations of dysphagia grade ≥ 2.

	**Self-training with logistic regression**		**Self-training with ridge regression**	
	Pseudolabels (incorrect)	Iterations	Pseudolabels (incorrect)	Iterations
710 labeled / 40 unlabeled	18 (1)	1	14 (0)	1
670 labeled / 40 unlabeled	19 (1)	1	14 (0)	1
630 labeled / 40 unlabeled	20 (1)	2	14 (0)	1
590 labeled / 40 unlabeled	20 (1)	2	14 (0)	1
550 labeled / 40 unlabeled	18 (1)	2	13 (0)	1
510 labeled / 40 unlabeled	18 (1)	1	14 (0)	1
470 labeled / 40 unlabeled	19 (1)	2	15 (0)	1
430 labeled / 40 unlabeled	20 (2)	2	16 (0)	1
390 labeled / 40 unlabeled	23 (4)	2	16 (0)	1
350 labeled / 40 unlabeled	20 (2)	2	17 (0)	1
310 labeled / 40 unlabeled	20 (2)	2	18 (1)	2
270 labeled / 40 unlabeled	20 (2)	2	16 (0)	1
230 labeled / 40 unlabeled	21 (2)	2	14 (0)	2

Table A9 | The total number of pseudolabeled observations (and the number of which are incorrect) and number of iterations in which new labels were added of the self-training method with logistic regression and ridge regression respectively, for each dataset with decreasing numbers of labeled observations of dysphagia grade ≥ 3.

	**Self-training with logistic regression**		**Self-training with ridge regression**	
	Pseudolabels (incorrect)	Iterations	Pseudolabels (incorrect)	Iterations
710 labeled / 40 unlabeled	29 (6)	1	22 (2)	1
670 labeled / 40 unlabeled	29 (6)	2	24 (3)	2
630 labeled / 40 unlabeled	29 (5)	2	25 (4)	2
590 labeled / 40 unlabeled	28 (6)	1	24 (3)	2
550 labeled / 40 unlabeled	29 (5)	2	24 (3)	1
510 labeled / 40 unlabeled	29 (5)	2	23 (2)	1
470 labeled / 40 unlabeled	28 (5)	2	24 (3)	1
430 labeled / 40 unlabeled	25 (3)	2	24 (3)	2
390 labeled / 40 unlabeled	25 (3)	2	24 (3)	1
350 labeled / 40 unlabeled	23 (3)	2	23 (3)	1
310 labeled / 40 unlabeled	22 (2)	2	23 (3)	1
270 labeled / 40 unlabeled	21 (2)	1	21 (2)	1
230 labeled / 40 unlabeled	21 (2)	1	22 (2)	1

Table A10 | The total number of pseudolabeled observations (and the number of which are incorrect) and number of iterations in which new labels were added with the self-training method with logistic regression for different confidence thresholds. The methods were applied to datasets with decreasing numbers of labeled observations of xerostomia grade ≥ 2.

	**Confidence threshold: 0.5**		**Confidence threshold: 0.6**		**Confidence threshold: 0.7**	
	Pseudolabels (incorrect)	Iterations	Pseudolabels (incorrect)	Iterations	Pseudolabels (incorrect)	Iterations
710 labeled / 40 unlabeled	40 (16)	1	19 (4)	1	9 (1)	2
670 labeled / 40 unlabeled	40 (16)	1	19 (4)	1	8 (1)	1
630 labeled / 40 unlabeled	40 (17)	1	19 (4)	1	8 (1)	2
590 labeled / 40 unlabeled	40 (18)	1	19 (4)	1	8 (1)	1
550 labeled / 40 unlabeled	40 (17)	1	19 (4)	1	8 (1)	1
510 labeled / 40 unlabeled	40 (16)	1	19 (4)	1	6 (0)	2
470 labeled / 40 unlabeled	40 (14)	1	19 (4)	1	5 (0)	1
430 labeled / 40 unlabeled	40 (16)	1	19 (4)	1	8 (1)	1
390 labeled / 40 unlabeled	40 (18)	1	19 (4)	2	12 (1)	2
350 labeled / 40 unlabeled	40 (18)	1	21 (6)	3	12 (1)	2
310 labeled / 40 unlabeled	40 (18)	1	18 (4)	1	12 (1)	2
270 labeled / 40 unlabeled	40 (16)	1	19 (4)	1	12 (1)	2
230 labeled / 40 unlabeled	40 (15)	1	20 (4)	2	12 (1)	2
190 labeled / 40 unlabeled	40 (15)	1	19 (4)	2	9 (1)	2
150 labeled / 40 unlabeled	40 (14)	1	28 (9)	5	14 (2)	2
110 labeled / 40 unlabeled	40 (14)	1	34 (13)	5	16 (2)	4
70 labeled / 40 unlabeled	40 (17)	1	32 (13)	2	18 (5)	6

Table A10 continued

	**Confidence threshold: 0.8**		**Confidence threshold: 0.9**		**Confidence threshold: 0.95**	
	Pseudolabels (incorrect)	Iterations	Pseudolabels (incorrect)	Iterations	Pseudolabels (incorrect)	Iterations
710 labeled / 40 unlabeled	5 (0)	1	3 (0)	1	0	0
670 labeled / 40 unlabeled	5 (0)	1	3 (0)	2	0	0
630 labeled / 40 unlabeled	4 (0)	1	1 (0)	1	0	0
590 labeled / 40 unlabeled	4 (0)	1	1 (0)	1	0	0
550 labeled / 40 unlabeled	4 (0)	1	0	0	0	0
510 labeled / 40 unlabeled	4 (0)	1	0	0	0	0
470 labeled / 40 unlabeled	4 (0)	1	0	0	0	0
430 labeled / 40 unlabeled	4 (0)	1	0	0	0	0
390 labeled / 40 unlabeled	4 (0)	1	0	0	0	0
350 labeled / 40 unlabeled	4 (0)	1	0	0	0	0
310 labeled / 40 unlabeled	5 (0)	2	0	0	0	0
270 labeled / 40 unlabeled	4 (0)	1	0	0	0	0
230 labeled / 40 unlabeled	5 (0)	1	0	0	0	0
190 labeled / 40 unlabeled	4 (0)	1	1 (0)	1	0	0
150 labeled / 40 unlabeled	5 (0)	1	0	0	0	0
110 labeled / 40 unlabeled	5 (0)	1	0	0	0	0
70 labeled / 40 unlabeled	4 (0)	1	0	0	0	0

Table A11 | The total number of pseudolabeled observations (and the number of which are incorrect) and number of iterations in which new labels were added of the self-training method with logistic regression and ridge regression. The methods were applied to datasets with decreasing ratios of labeled vs unlabeled observations of xerostomia grade ≥ 2.

	**Self-training with logistic regression**		**Self-training with ridge regression**	
	Pseudolabels (incorrect)	Iterations	Pseudolabels (incorrect)	Iterations
710 labeled / 40 unlabeled	10 (0)	1	10 (0)	1
670 labeled / 80 unlabeled	15 (0)	1	14 (0)	1
630 labeled / 120 unlabeled	24 (0)	1	24 (0)	2
590 labeled / 160 unlabeled	37 (0)	2	36 (0)	2
550 labeled / 200 unlabeled	48 (3)	2	46 (2)	2
510 labeled / 240 unlabeled	58 (5)	2	56 (5)	3
470 labeled / 280 unlabeled	81 (8)	6	64 (4)	2
430 labeled / 320 unlabeled	102 (13)	5	95 (11)	7
390 labeled / 360 unlabeled	124 (19)	5	113 (15)	6
350 labeled / 400 unlabeled	130 (18)	4	121 (15)	7
310 labeled / 440 unlabeled	141 (21)	4	123 (16)	7
270 labeled / 480 unlabeled	149 (23)	5	138 (19)	4
230 labeled / 520 unlabeled	166 (27)	4	153 (21)	8
190 labeled / 560 unlabeled	203 (39)	7	174 (27)	5
150 labeled / 600 unlabeled	319 (79)	19	187 (30)	5

(a)

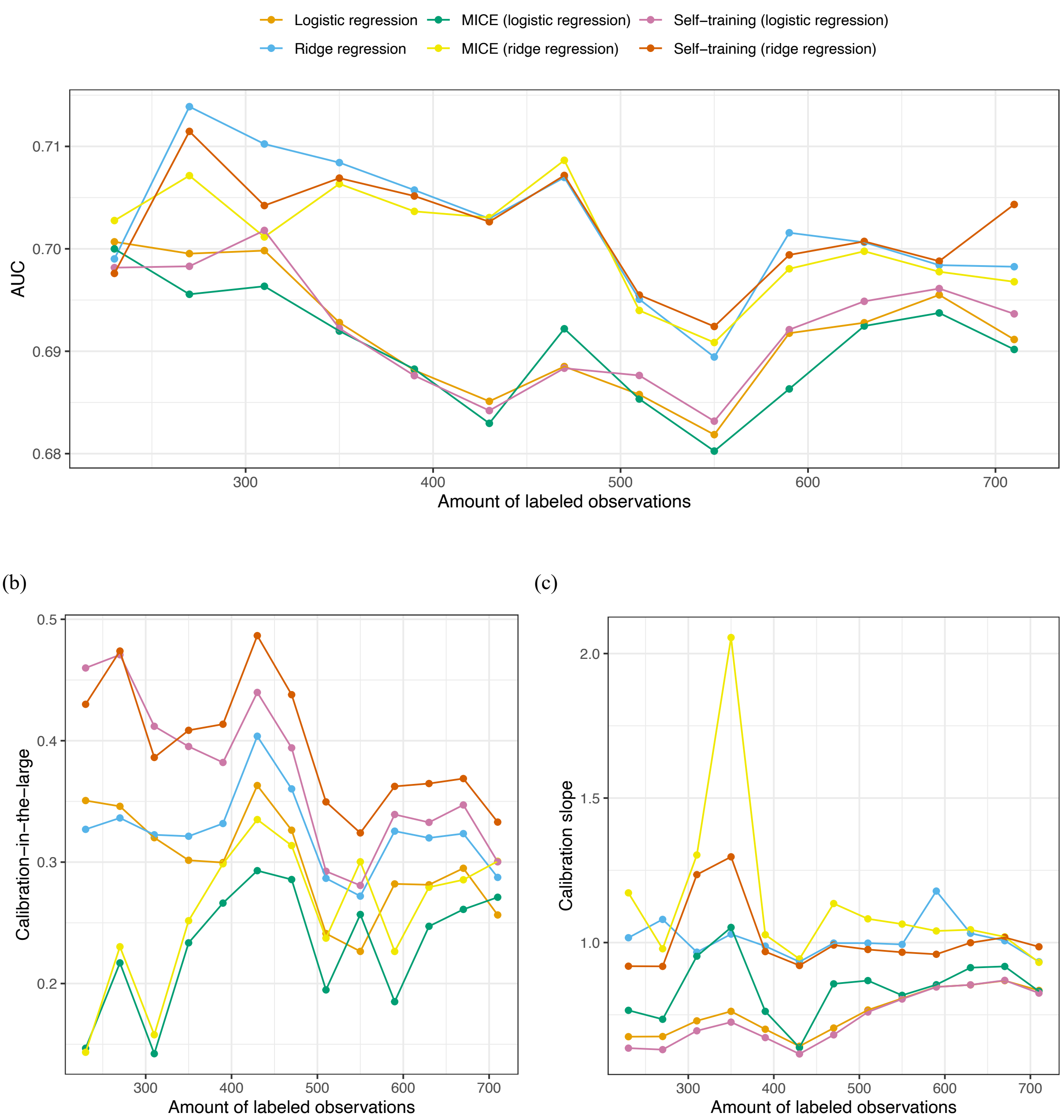

Figure A1 | External validation of the models for xerostomia grade ≥ 3 for different amounts of labeled data. The amount of unlabeled data is fixed at 40 observations. (a) The AUCs, (b) the calibration intercepts, and (c) the calibration slopes.

(a)

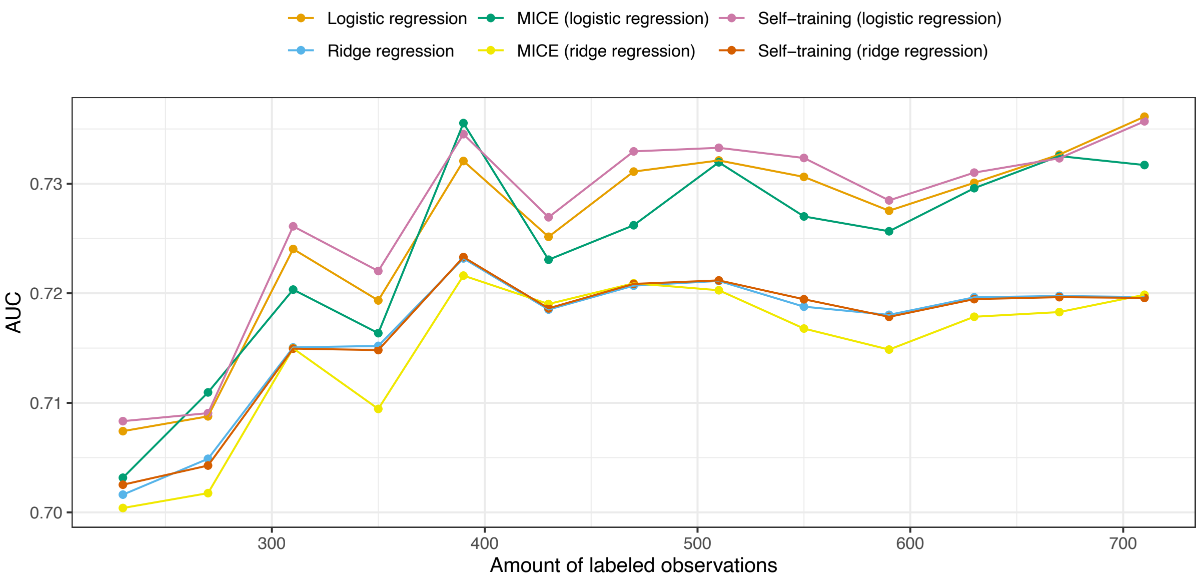

(b)

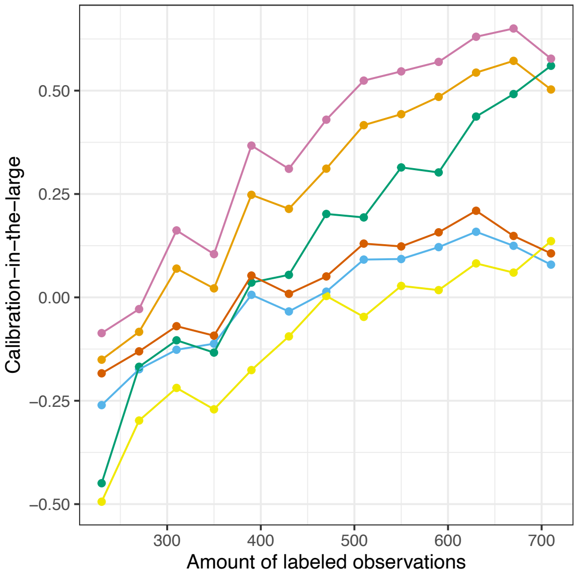

(c)

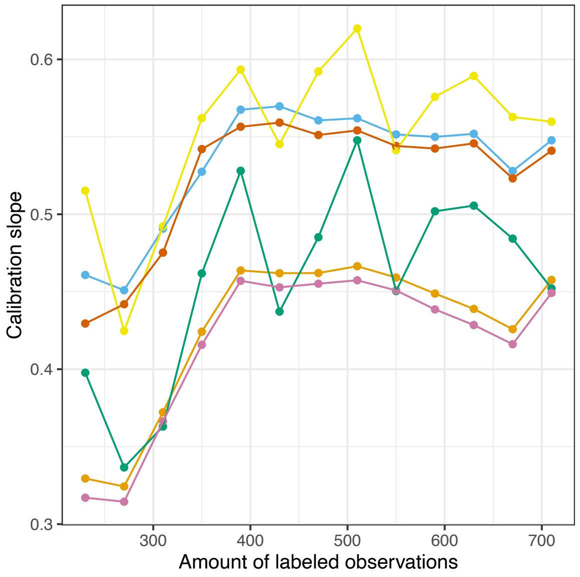

Figure A2 | External validation of the models for dysphagia grade ≥ 3 for different amounts of labeled data. The amount of unlabeled data is fixed at 40 observations. (a) The AUCs, (b) the calibration intercepts, and (c) the calibration slopes.

(a) Logistic regression

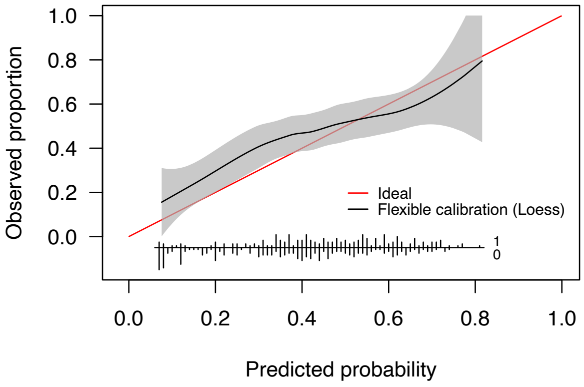

(b) Ridge regression

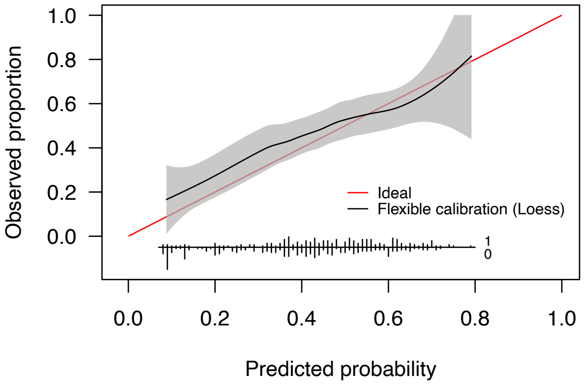

(c) Logistic regression after MICE

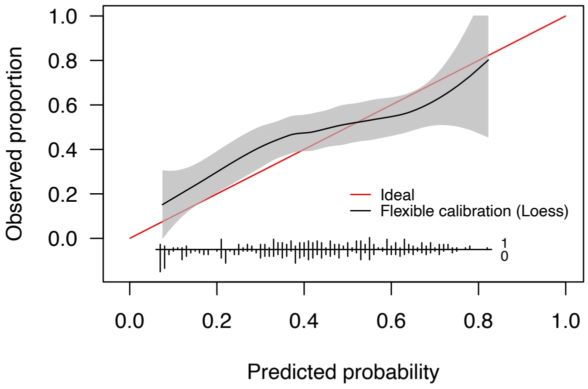

(d) Ridge regression after MICE

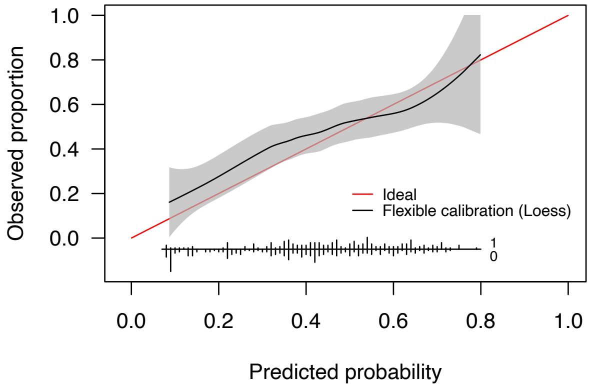

(e) Self-training with logistic regression as classifier

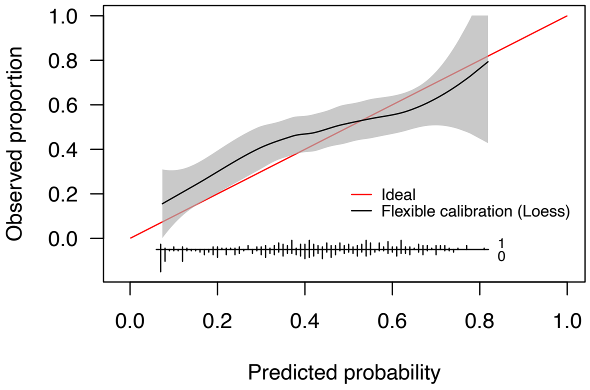

(f) Self-training with ridge regression as classifier

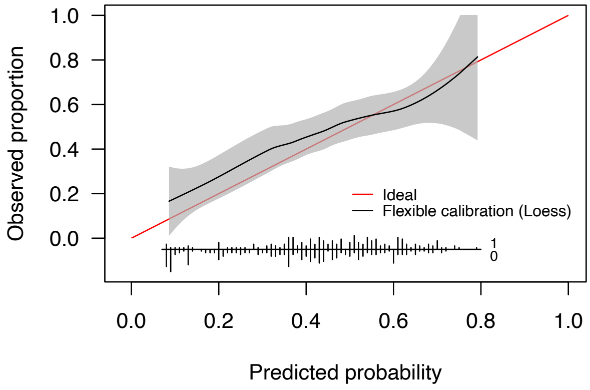

Figure A3 | Calibration curves of the xerostomia grade ≥ 2 models. (a) Logistic regression, (b) Ridge regression, (c) Logistic regression after multiple imputation of the outcome with MICE, (d) Ridge regression after multiple imputation of the outcome with MICE, (e) Self-training with logistic regression as classifier, (f) Self-training with ridge regression as classifier.

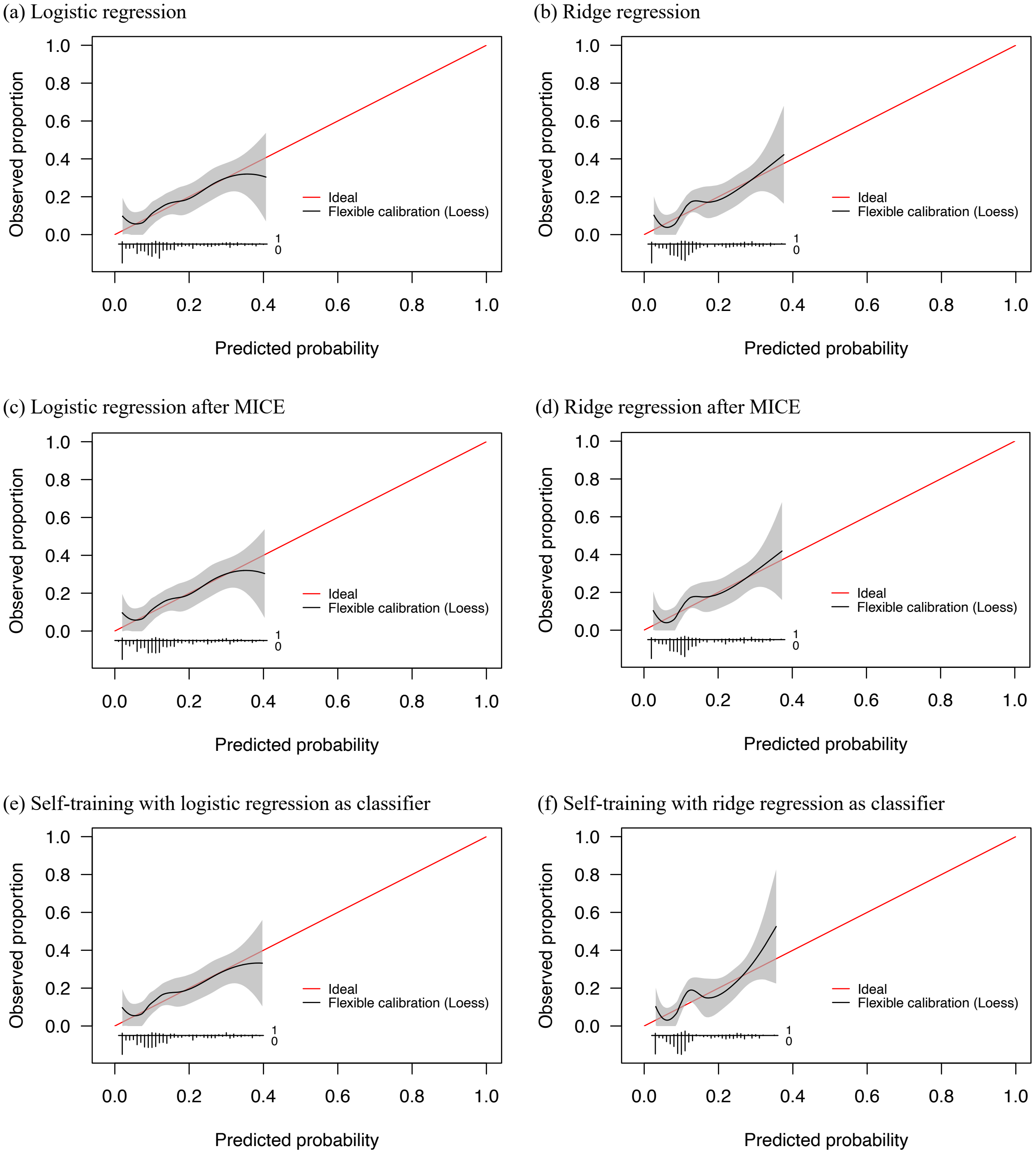

Figure A4 | Calibration curves of the xerostomia grade ≥ 3 models. (a) Logistic regression, (b) ridge regression, (c) logistic regression after multiple imputation of the outcome with MICE, (d) ridge regression after multiple imputation of the outcome with MICE, (e) self-training with logistic regression as classifier, (f) self-training with ridge regression as classifier.

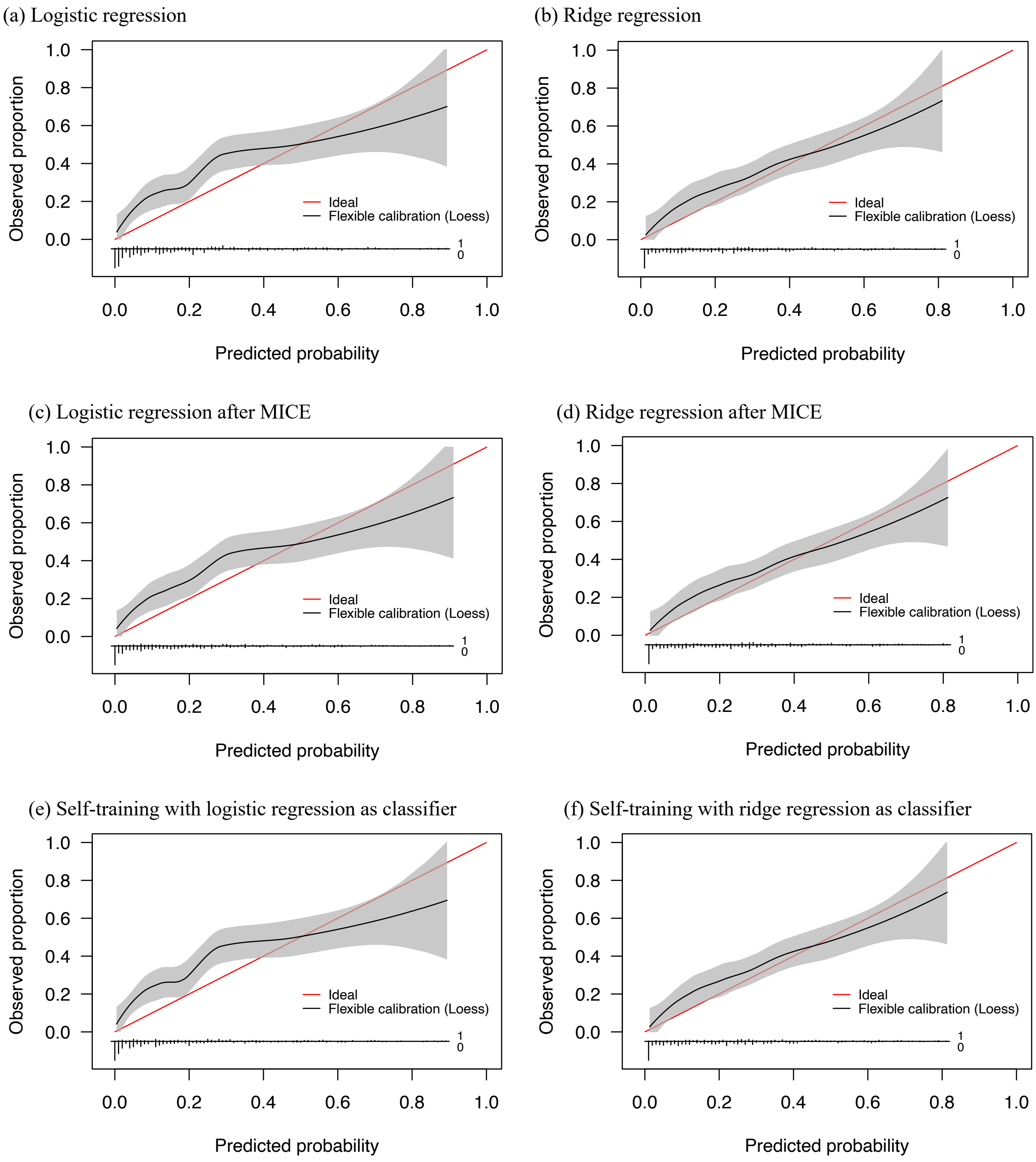

Figure A5 | Calibration curves of the dysphagia grade ≥ 2 models. (a) Logistic regression, (b) ridge regression, (c) logistic regression after multiple imputation of the outcome with MICE, (d) ridge regression after multiple imputation of the outcome with MICE, (e) self-training with logistic regression as classifier, (f) self-training with ridge regression as classifier.

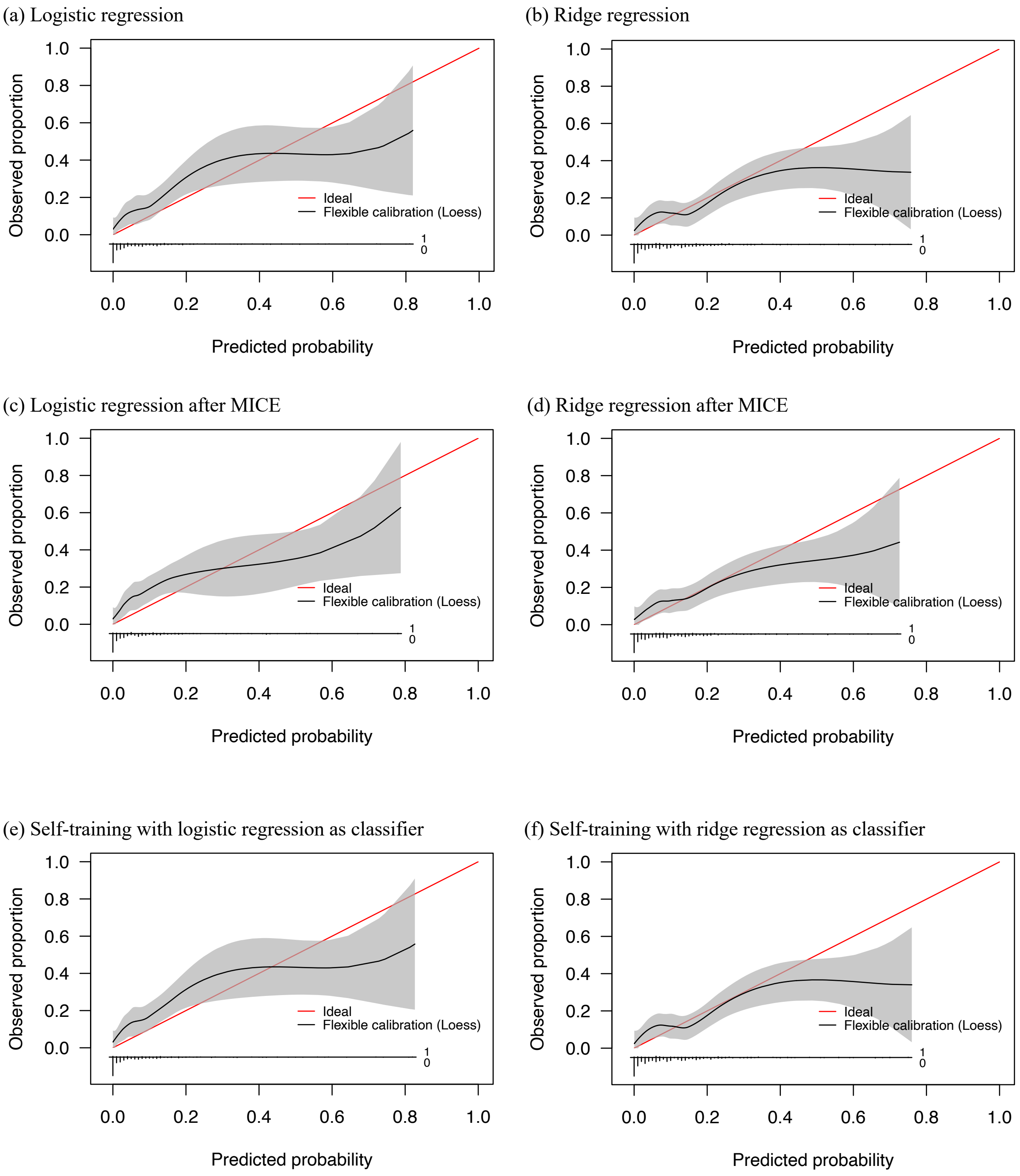

Figure A6 | Calibration curves of the dysphagia grade ≥ 3 models. (a) Logistic regression, (b) ridge regression, (c) logistic regression after multiple imputation of the outcome with MICE, (d) ridge regression after multiple imputation of the outcome with MICE, (e) self-training with logistic regression as classifier, (f) self-training with ridge regression as classifier.

(a)

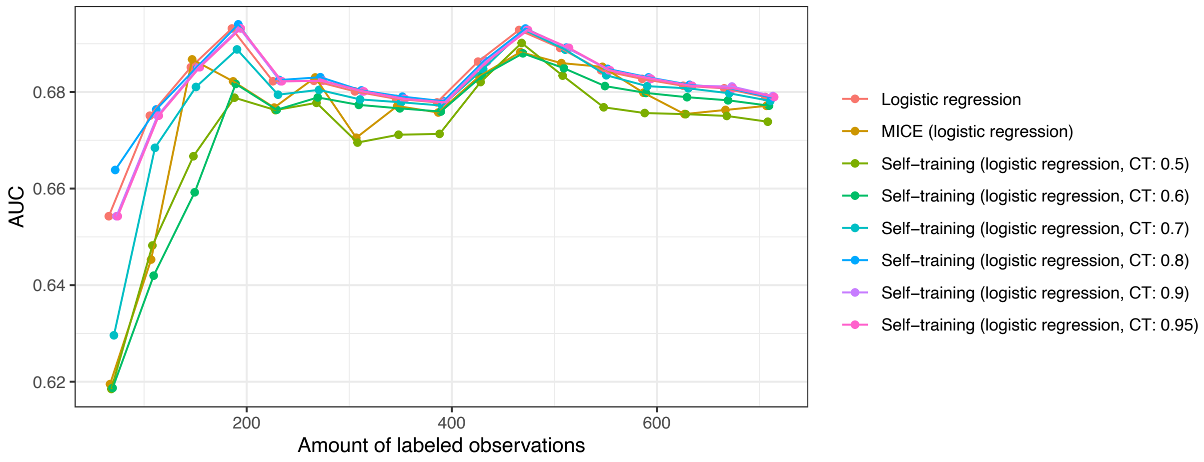

(b)

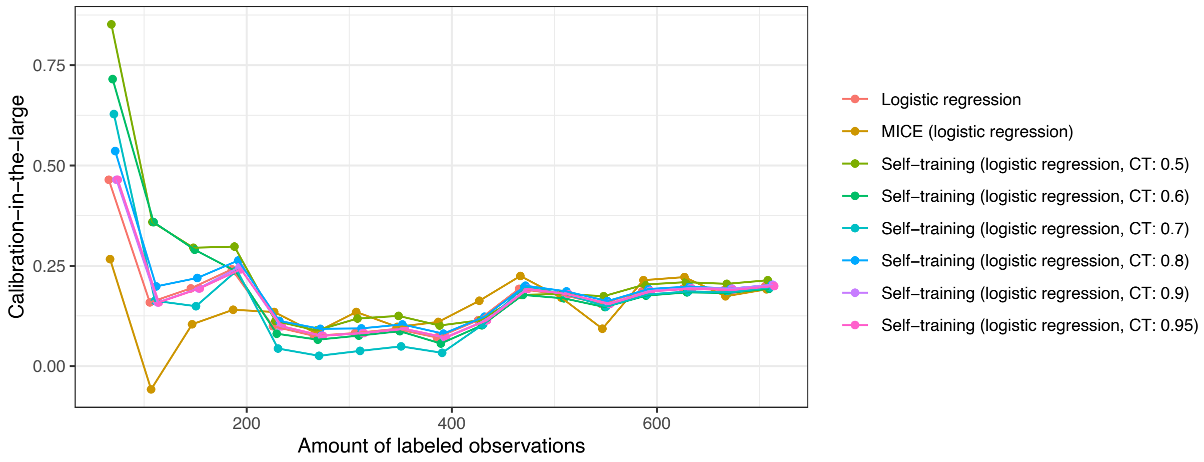

(c)

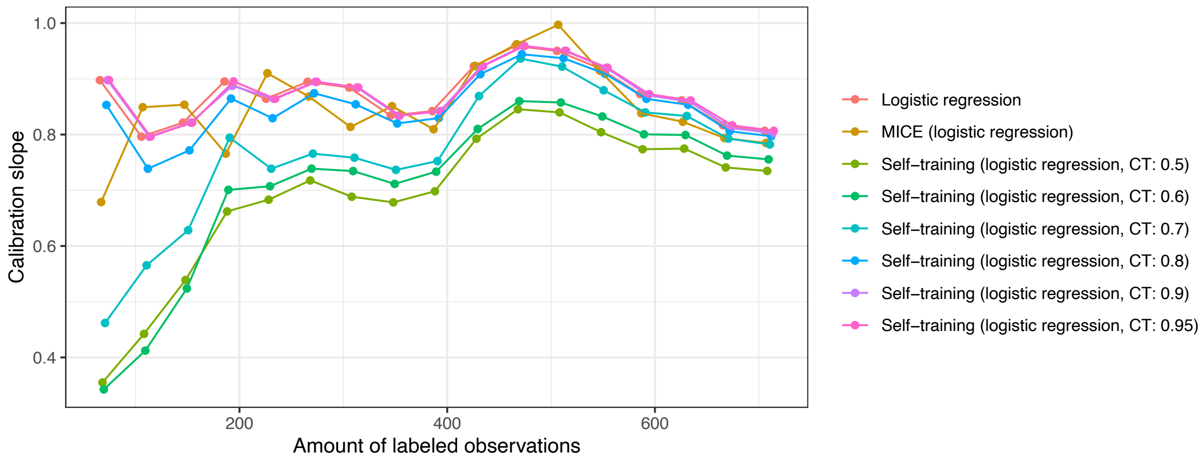

Figure A7 | External validation of the xerostomia grade ≥ 2 models with different confidence thresholds (CTs) for the self-training method with logistic regression. The x-axis shows the decrease in the amount of labeled data. The amount of unlabeled data is fixed at 40 observations. (a) The AUCs, (b) the calibration-in-the-large, and (c) the calibration slopes.

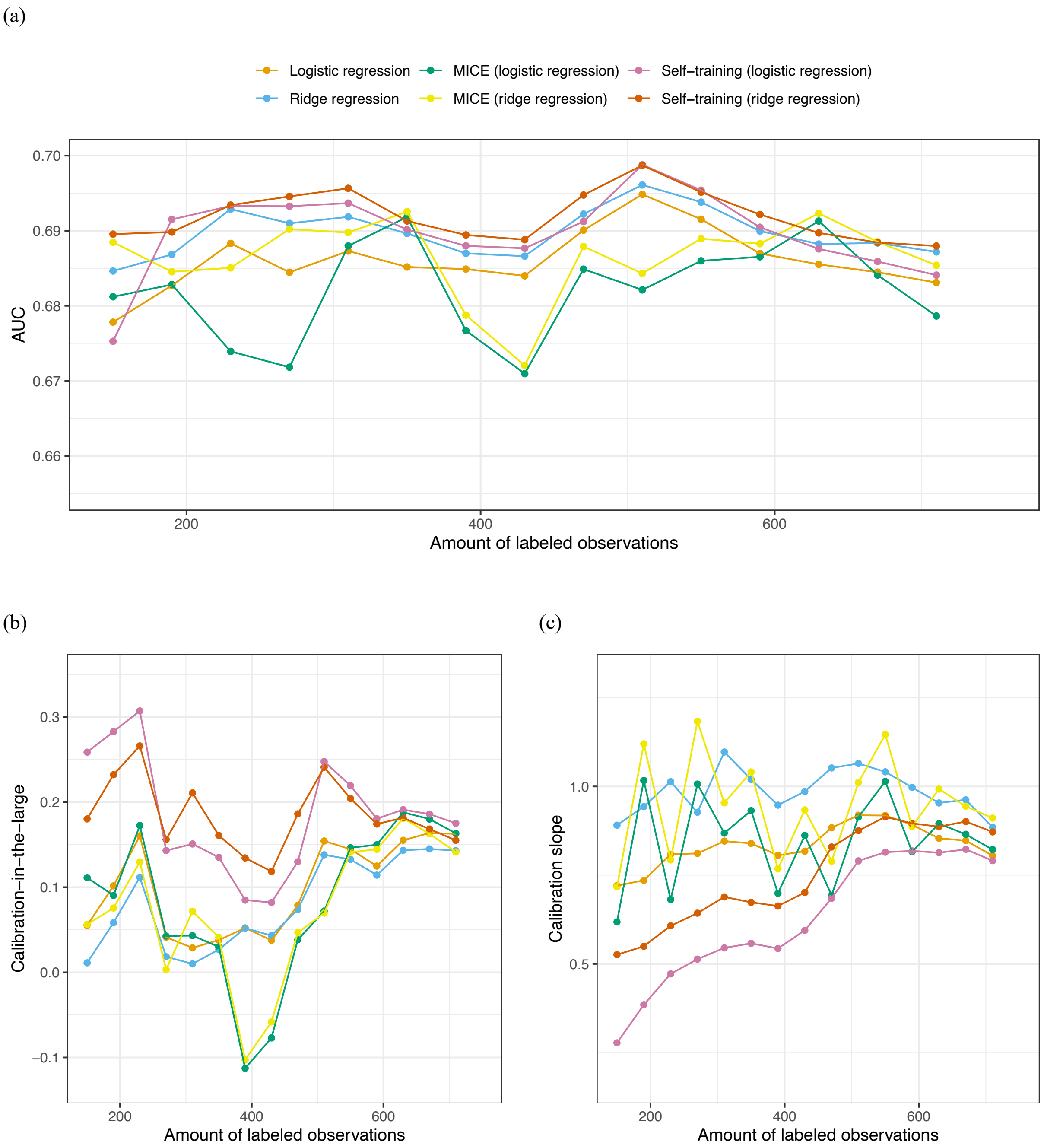

Figure A8 | External validation of the xerostomia grade ≥ 2 models with decreasing proportions of unlabeled data. The x-axis shows the amount of labeled data, while the total amount of observations (labeled plus unlabeled data) remains 750. (a) The AUCs, (b) the calibration-in-the-large, and (c) the calibration slopes.

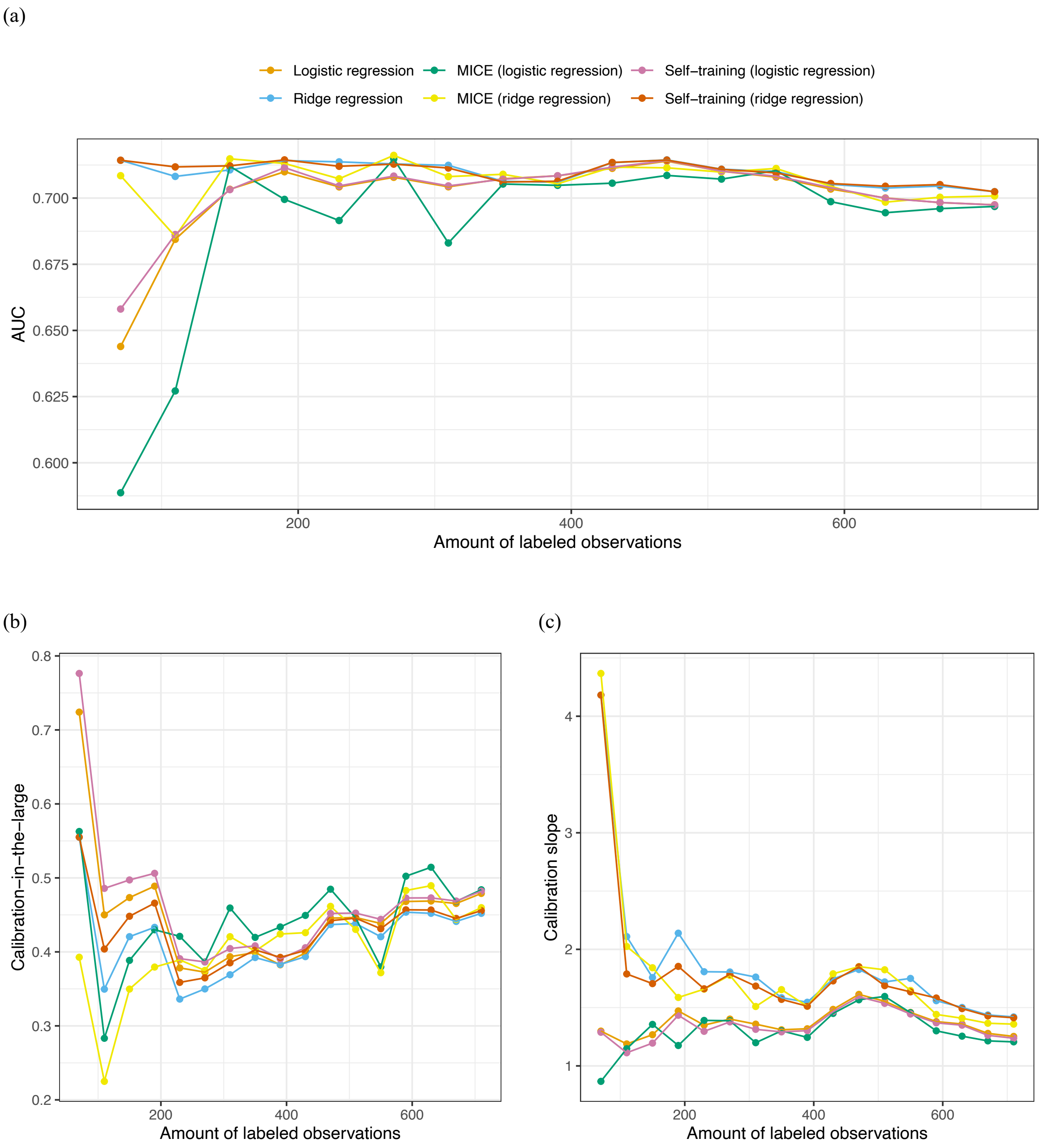

Figure A9 | External validation on the UMCG cohort of the models for xerostomia grade ≥ 2 for different amounts of labeled data. The amount of unlabeled data is fixed at 40 observations. (a) The AUCs, (b) the calibration intercepts, and (c) the calibration slopes.

(a)

(b) (c)

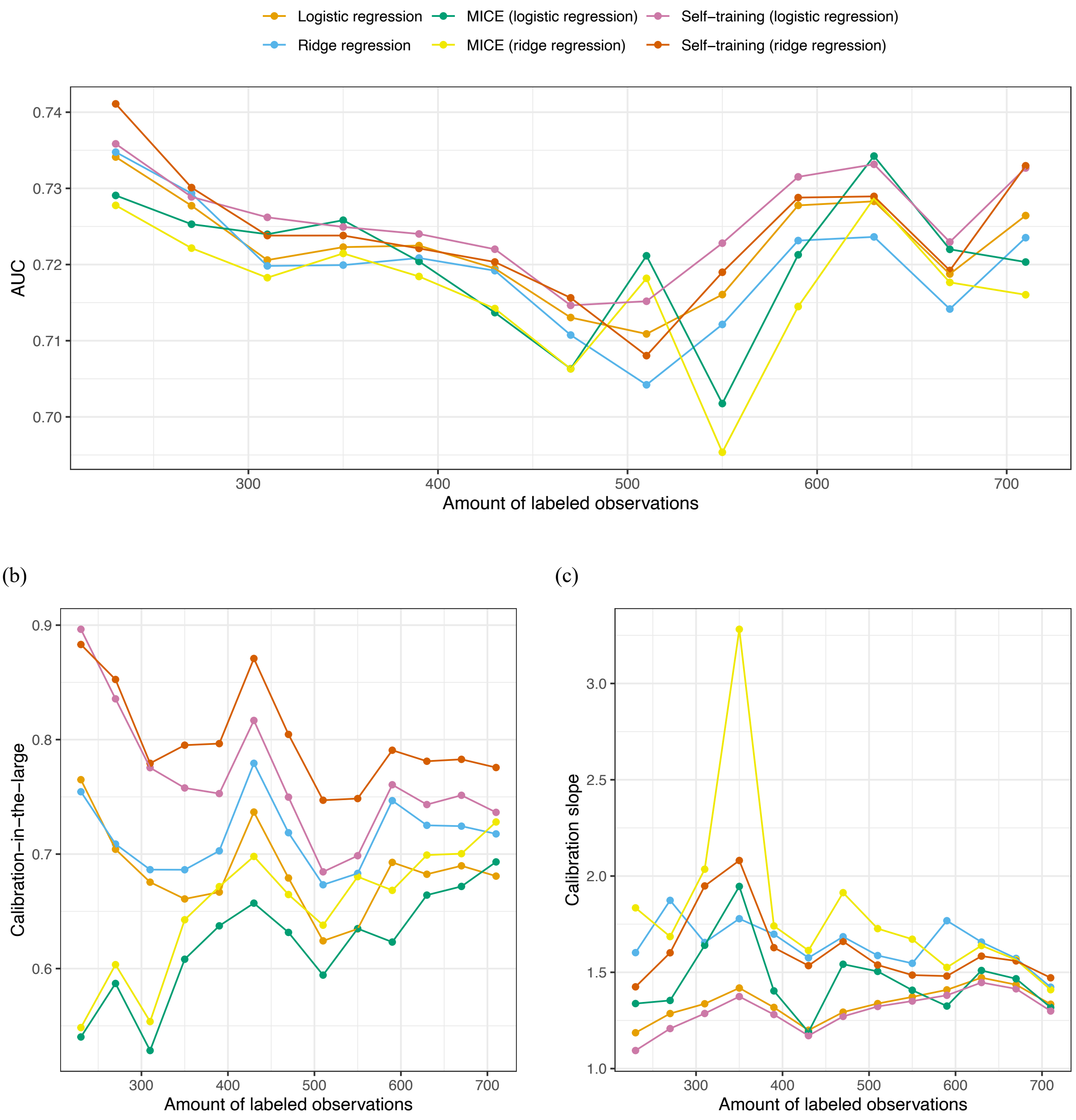

Figure A10 | External validation on the UMCG cohort of the models for xerostomia grade ≥ 3 for different amounts of labeled data. The amount of unlabeled data is fixed at 40 observations. (a) The AUCs, (b) the calibration intercepts, and (c) the calibration slopes.

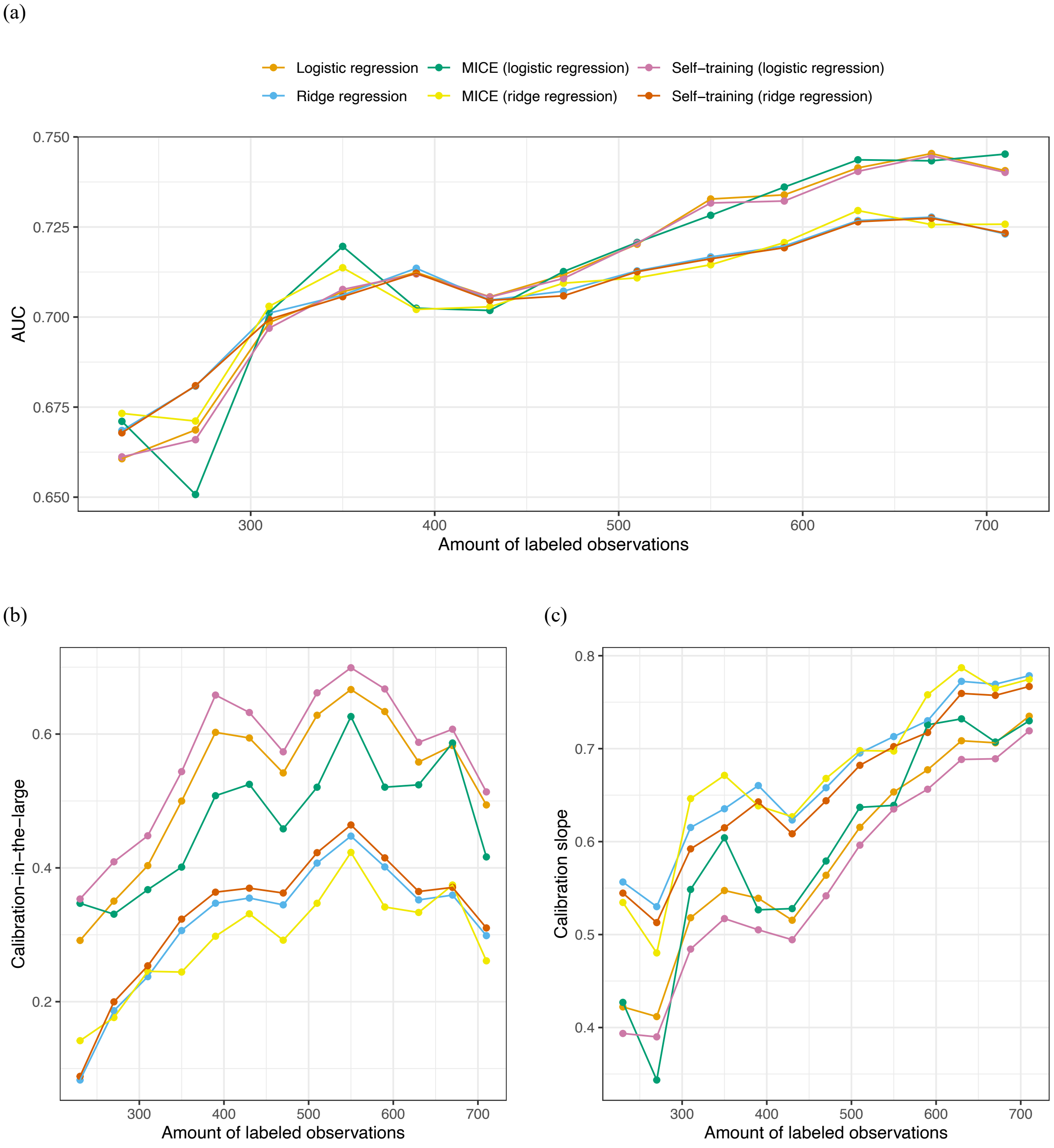

Figure A11 | External validation on the UMCG cohort of the models for dysphagia grade ≥ 2 for different amounts of labeled data. The amount of unlabeled data is fixed at 40 observations. (a) The AUCs, (b) the calibration intercepts, and (c) the calibration slopes.

(a)

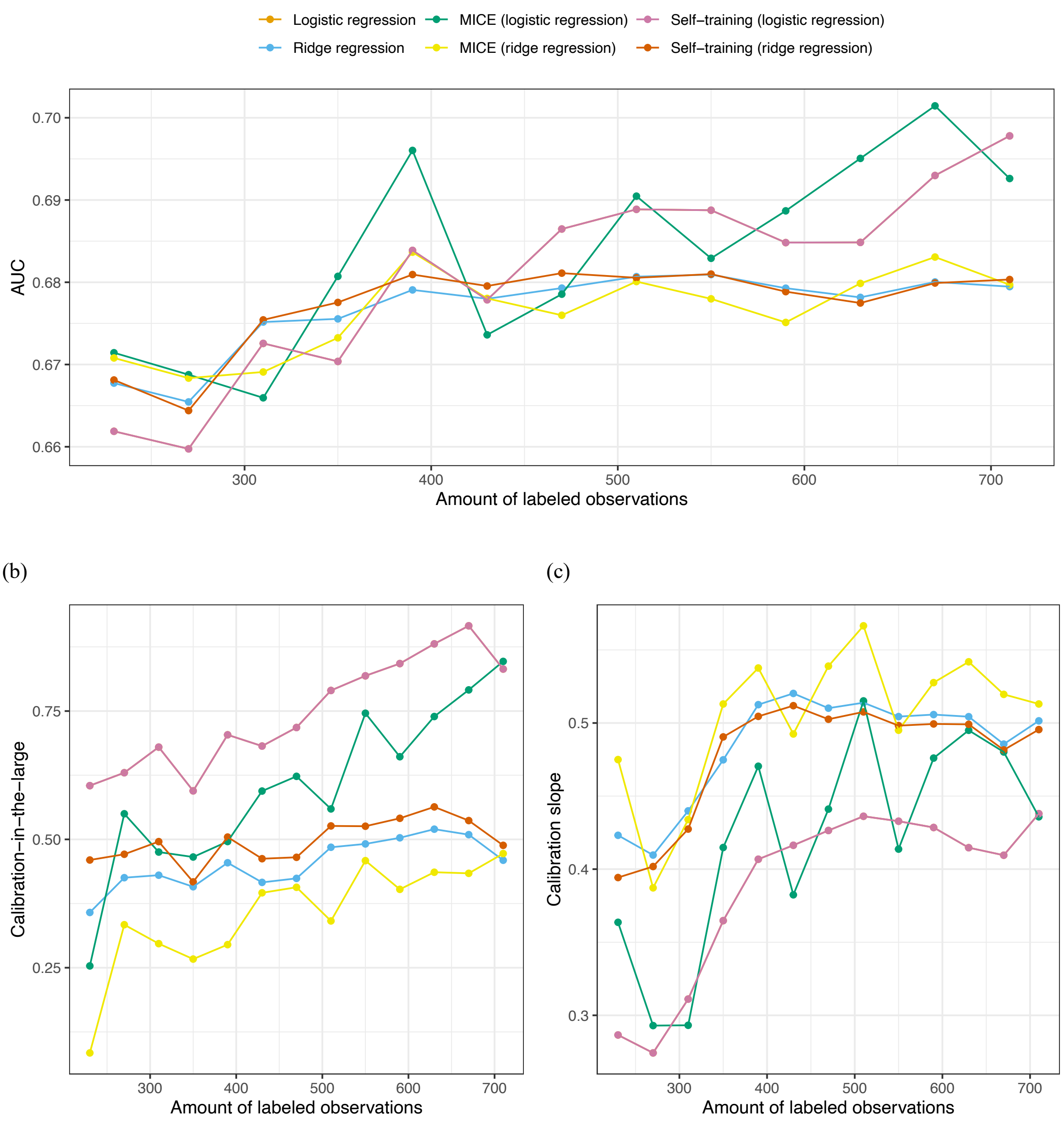

Figure A12 | External validation on the UMCG cohort of the models for dysphagia grade ≥ 3 for different amounts of labeled data. The amount of unlabeled data is fixed at 40 observations. (a) The AUCs, (b) the calibration intercepts, and (c) the calibration slopes.

Appendix B

Details on implementation of self-training

The semi-supervised method of self-training was used to examine whether the inclusion of unlabeled data by semi-supervised learning would improve NTCP modeling. Besides the decision on the classifier or learner to include (here logistic and ridge regression), a number of additional decisions to design a self-training model have to be made, such as the confidence threshold, and the stopping criterion after which no more iterations take place (Triguero *et al.*, 2015)[1]. The confidence threshold determines the probabilities for the unlabeled outcomes that are certain enough to pseudolabel the outcomes; when the probability is larger than the threshold, the outcome is pseudolabeled as having the event, while outcomes with a probability smaller than 1 minus the threshold are labeled as not having the event. Outcomes that do not meet the threshold remain unlabeled in the respective iteration. The confidence threshold was set at an intermediate value of 0.8 (following Soares *et al.* (2016)[2] in self-training models for xerostomia prediction). In this way, the threshold would be low enough to be reached and thereby to add pseudolabels, but still high enough to prevent the addition of large amounts of wrongly classified pseudolabels. The process of model development and using the predictions to add labels was iterated until it reached a stopping criterion, which was determined to be when no unlabeled data were left or after a maximum of 50 iterations. The R code to conduct the experiments with the self-training method is available at https://github.com/isa-sp/semi-supervised_NTCP.

[1] Triguero, I., García, S., & Herrera, F. (2015). Self-labeled techniques for semi-supervised learning: Taxonomy, software and empirical study. *Knowledge and Information Systems*, 42(2), 245–284.

[2] Soares, I., Dias, J., Rocha, H., Khouri, L., Carmo Lopes, M. D., & Ferreira, B. (2016). Semi-supervised self-training approaches in small and unbalanced datasets: Application to xerostomia radiation side-effect. In *XIV Mediterranean Conference on Medical and Biological Engineering and Computing 2016* (pp. 828-833). Springer, Cham.
